# Extreme Values of Hemoglobin A1c Are Associated With Increased Risks of Chronic Obstructive Pulmonary Disease in Patients With Type 2 Diabetes: A Competing Risk Analysis in National Cohort of Taiwan Diabetes Study

**DOI:** 10.1097/MD.0000000000000367

**Published:** 2015-01-09

**Authors:** Chia-Ing Li, Tsai-Chung Li, Chiu-Shong Liu, Wen-Yuan Lin, Ching-Chu Chen, Sing-Yu Yang, Cheng-Chieh Lin

**Affiliations:** From the Department of Medical Research (C-IL, C-SL, C-CL), China Medical University Hospital; School of Medicine (C-IL, C-SL, W-YL, C-CL), College of Medicine; Graduate Institute of Biostatistics (T-CL, S-YY), College of Management, China Medical University; Department of Healthcare Administration (T-CL), College of Health Science, Asia University; Department of Family Medicine (C-SL, C-CL, W-YL); Division of Endocrinology and Metabolism (C-CC), Department of Medicine, China Medical University Hospital; and School of Chinese Medicine (C-CC), College of Chinese Medicine, China Medical University, Taichung, Taiwan.

## Abstract

The purpose of this study was to evaluate the relationship between glycated hemoglobin (HbA1c) and chronic obstructive pulmonary disease (COPD) in patients with type 2 diabetes.

We conducted a retrospective cohort study involving 45,753 patients with type 2 diabetes, who participated in the National Diabetes Case Management Program in Taiwan. HbA1c at baseline and COPD events over the subsequent years were analyzed.

After multivariate adjustment, the COPD risk increased among patients with HbA1c levels <6.0%, compared with that in patients with HbA1c levels ranging from 6.0% to 7.0% (hazard ratio: 1.19, 95% confidence interval (CI): 1.06–1.34). Similarly, high HbA1c levels (≥10%) were independently associated with COPD (1.19, 95% CI: 1.06–1.32). A U-shaped relationship was observed between HbA1c levels and COPD incidence.

HbA1c levels lower than 6.0% and higher than 10% are associated with an increased risk of COPD in patients with type 2 diabetes. These findings suggest that meeting the recommended HbA1c targets might reduce the risk of COPD, but care should be taken not to pose risks to this population.

## INTRODUCTION

The prevalence of diabetes has dramatically increased in both developed and developing countries in the previous decades. Type 2 diabetes has become an imperative public health challenge among the Chinese population, particularly in Taiwan, mainland China, Hong Kong, and Singapore, which together account for at least one-fifth of the global population.^1–4^ The increase of type 2 diabetes is due to the prevalence of a sedentary lifestyle and a high-energy dietary intake. In addition, diabetes and its complications represent 1 in every 4 hospital beds.^5^

Plasma glycated hemoglobin (HbA1c) levels, which reflect the mean ambient fasting and postprandial glycaemia over a 2- to 3-month period, are the most widely used marker of long-term glycoregulation.^6^ HbA1c characterizes dysglycemia by the slow, irreversible, and nonenzymatic glycation of valine and lysine residues in the hemoglobin molecule.^7^ It is at the center of the clinical management of hyperglycemia, and is considered to be a valuable monitoring tool in treating diabetic patients, because the corresponding test is easier to perform than an oral glucose tolerance test, and is independent of the prandial status of the patient.^8^ Compared with fasting glucose, HbA1c has been treated as an improved long-term glycemic control marker because it can be tested in a nonfasting status, and is a stable marker of glucose levels. The HbA1c level is also widely recommended as a therapeutic guideline for preventing cardiovascular complications in diabetic patients.^9^

Currently, much attention has been focused on lung dysfunction in diabetes, emphasizing the emerging potential clinical implications of such dysfunction.^10^ Decreased lung elastic recoil,^11^ as well as pulmonary vascular changes generated by a lowered carbon monoxide (CO) transfer capacity resulting from a decreased pulmonary capillary blood volume,^12^ has been observed in young patients with type 1 diabetes who were lifelong nonsmokers without allergies or lung disease. These findings suggest that the lung may be a target organ for diabetic microangiopathy. It has been suggested that patients with diabetes are at risk for developing chronic airflow obstructions that could subsequently incur disability because of the mechanical dysfunction of the lungs and airways. The noted evidence demonstrated that physiological abnormalities were modest in degree in patients with diabetes.^13^ To our knowledge, no previous studies have examined the relationship between plasma HbA1c levels and the incidence of chronic obstructive pulmonary disease (COPD) in patients with type 2 diabetes. Additional data are needed to characterize the effects of HbA1c levels on COPD. We analyzed the relationship between the COPD risk and HbA1c levels in Chinese patients with type 2 diabetes, who participated in the National Diabetes Case Management Program (NDCMP) in Taiwan.

## METHODS

### Study Population

**FIGURE 1:**
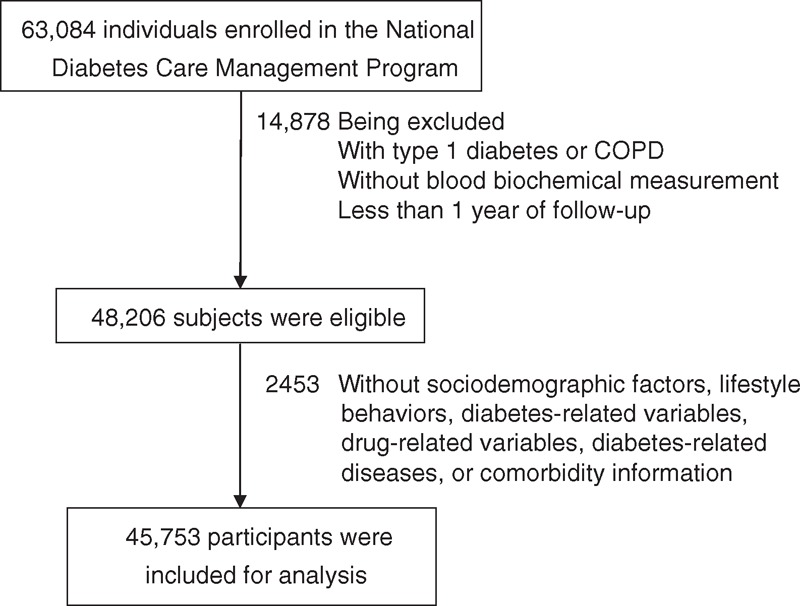
Flowchart of recruitment procedures for the current study. COPD = chronic obstructive pulmonary disease.

We conducted a retrospective cohort study, the Taiwan Diabetes Cohort Study, among all enrollees in the NDCMP in Taiwan. The Taiwan Diabetes Cohort Study is a population-based cohort study of 63,084 ethnic Chinese patients with type 2 diabetes who were enrolled in the NDCMP, a case management program set up by the National Health Insurance (NHI) Bureau in Taiwan during 2002 to 2004. The entry date into the NDCMP was defined as the index date. All patients with a clinically confirmed diagnosis of diabetes mellitus (DM) based on the American Diabetes Association (ADA) criteria (International Classification of Disease, 9th Revision, Clinical Modification [ICD-9-CM] diagnosis code 250) were invited to enroll in this program. Patients with type 1 diabetes (ICD-9-CM code 250.×1/×3), gestational diabetes (ICD-9-CM code 648.83), and COPD, as well as those <30 years, were excluded. All patients who could provide at least 1 year of follow-up for estimating the COPD incidence and could provide at least 1 HbA1c measurement were included in the study. These inclusion criteria were met by 48,206 continuously enrolled diabetic patients. After excluding patients with missing data for sociodemographic factors, lifestyle behaviors, diabetes-related variables, drug-related variables, diabetes-related diseases, or comorbidity information, 45,753 patients were included in the data analysis (Figure 1). The current study was approved by the Ethical Review Board of the China Medical University Hospital (CMUH102-REC1–035).

### Data Sources for Baseline and Follow-Up Assessments

In the current study, datasets of inpatient care (admissions) and outpatient care (visits) during 2002 to 2011 were used. Every person in Taiwan has a unique personal identification number (PIN). For security and privacy purposes, data on patient identities are cryptographically scrambled by the National Health Insurance Research Database (NHIRD). All NHI datasets can be interlinked with the scrambled PIN of each individual. Claims data are randomly audited by the insurance system. Expert reviews on a random sample for every 50 to 100 ambulatory and inpatient claims in each hospital and clinic are conducted quarterly to enhance the validity of the claims data. A severe penalty is placed on every false diagnostic report by the NHI Bureau. Data consist of information on demographic characteristics, date and source of diagnosis, ambulatory care, inpatient admission, and outpatient and inpatient treatment for all insured people. The ICD-9-CM is used to identify the individual health status. Because of the comprehensive coverage of the NHI program, the proportion of enrollees withdrawing from this program is considerably low. Thus, bias resulting from loss to follow-up is negligible.

The NDCMP is a program that has been conducted by the Ministry of Health and Welfare of Taiwan since 2001. The main aim of this program is to increase the quality of diabetes care by increasing the frequency of monitoring, providing continuity of care, and decreasing diabetes-related complications. When entering the NDCMP, enrollees undergo a comprehensive assessment of the status of their disease and complications, as well as a series of blood tests, urine tests, and body measurements. In addition, participants are required to complete a standardized and computerized questionnaire administered by a case management nurse to record the previous or current status of their disease, medication, and lifestyle behaviors. Blood is drawn from an antecubital vein in the morning, after a 12-hour overnight fast, and sent for analysis within 4 hours after collection.

### Urbanization Level

Level of urbanization was determined by the residential areas of the study subjects, which belong to 365 Taiwan townships. There were 7 urbanization levels, which correspond to high-density urban area, medium-density urban area, newly developed area, general area, aging society area, rural area, and nondeveloped area (seclusion area).^14^ The variables used for urbanization level consist of population ratio of people with college or above educational levels, population density (people/km^2^), population ratio of agricultural workers, population ratio of elder people >65 years old, the number of physicians per 100,000 people, etc.

### Outcome Ascertainment

COPD was determined from inpatient and outpatient claims data, based on major discharge diagnosis codes from the ICD-9 (ICD-9-M codes 490–496). We searched the NHIRD database for this population to identify patients who had at least 1 inpatient claim or at least 3 outpatient claims. By linking the unique identification number with this computerized file, 4322 patients suffering from COPD within 8 years of follow-up were identified from this cohort. The cohort was followed up from the index date to December 31, 2011 or until a COPD event, death, or withdrawal from the insurance occurred. Data on other chronic medical conditions were collected for the 12-month period preceding cohort entry, using outpatient and inpatient claims data. The histories of coronary artery disease (ICD-9-CM codes 410–413, 414.01–414.05, 414.8, and 414.9), congestive heart failure (ICD-9-CM codes 428, 398.91, and 402.×1), cancer (ICD-9-CM codes 140–149, 150–159, 160–165, 170–175, 179–189, 190–199, 200, 202, 203, 210–213, 215–229, 235–239, 654.1, 654.10, 654.11, 654.12, 654.13, and 654.14), atrial fibrillation (ICD-9-CM code 427.31), stroke (ICD-9-CM codes 430–438), hyperlipidemia (ICD-9-CM code 272), hypertension (ICD-9-CM codes 401–405), and chronic hepatitis (ICD-9-CM codes 571, 572.2, 572.3, 572.8, 573.1, 573.2, 573.3, 573.8, and 573.9) were identified as comorbidities before the index date.

### Statistical Analysis

For each individual patient, the baseline HbA1c measurement was determined from datasets of electronic lab records. The groups of patients exhibited HbA1c levels of <6%, 6%–7%, 7%–8%, 8%–9%, 9%–10%, and ≥10% from the baseline. The extended Cox proportional hazards model using the Lunn–McNeil approach was applied as a modified Cox proportional hazards model that considered competing risks and allowed for multivariable adjustment. The extended Cox proportional hazard models with the competing risk of death were used to evaluate the association between HbA1c level and incident COPD. We calculated the hazard ratios (HRs) and their 95% confidence intervals (CIs) by adjusting for age, gender, and multiple variables. The multivariate model was adjusted for age (continuous), gender (male, female), smoking (yes, no), alcohol consumption (yes, no), duration of diabetes (continuous), type of hypoglycemic drug (no medication, metformin monotherapy, sulfonylurea monotherapy, oral antidiabetic drug [OAD] monotherapy other than metformin or sulfonylurea [OAD-other], combination of metformin plus sulfonylurea, combination of metformin plus OAD-other, combination of sulfonylurea plus OAD-other, combination of 2 OAD-other, combination of 3 OAD, combination of more than 3 OAD, insulin monotherapy, insulin plus 1 OAD, insulin plus more than 1 OAD, and short-time OAD; OAD-other consisted of α-glucosidase inhibitors, thiazolidinedione, and meglitinide), hospitalization status 1 year prior to the baseline (yes, no), hypertension (yes, no), antihypertensive treatment (yes, no), obesity (yes, no), coronary artery disease (yes, no), congestive heart failure (yes, no), cancer (yes, no), hyperlipidemia (yes, no), atrial fibrillation (yes, no), stroke (yes, no), and chronic hepatitis (yes, no). We calculated *P* for evaluating the trend across the baseline HbA1c categories, and tested the proportionality assumption by including an interaction term for baseline HbA1c categories using person-time in the Cox models. No statistically significant violation was found. All analyses were performed using SAS version 9.3 (SAS Institute, Cary, NC). All *P*-values were 2-tailed, and *P* < 0.05 was considered to indicate statistical significance.

## RESULTS

**FIGURE 2:**
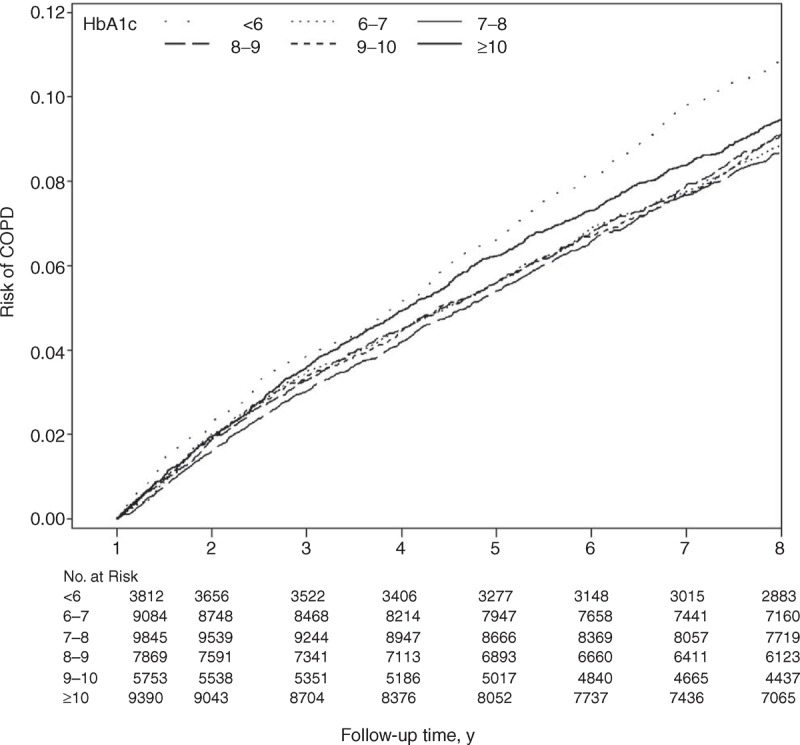
Risks of COPD for different HbA1c. COPD = chronic obstructive pulmonary disease, HbA1c = glycated hemoglobin.

During an average of 7.9 years of follow-up, a total of 4322 incidence cases of COPD were identified in diabetic patients, corresponding to a crude incidence rate of 11.90/1000 person-years (13.28 for men and 10.69 for women). Table 1  plots the baseline sociodemographic and clinical factors of the patients, grouped according to COPD status. COPD patients were more likely to be male, to have a greater mean age and duration of diabetes, to use tobacco, insulin injections, or insulin injections plus oral hypoglycemic drugs, hypertension drug treatment, and to suffer from coronary artery disease, congestive heart failure, hypertension, atrial fibrillation, or hypoglycemia (Table 1 ) than other patients. Figure 2 presents the Kaplan–Meier cumulative incidence curves of COPD, within the subgroups defined by HbA1c levels. Patients with HbA1c levels <6% or ≥10% exhibited a higher risk (log-rank *P* < 0.01) of developing COPD than did other patients.

**TABLE 1:**
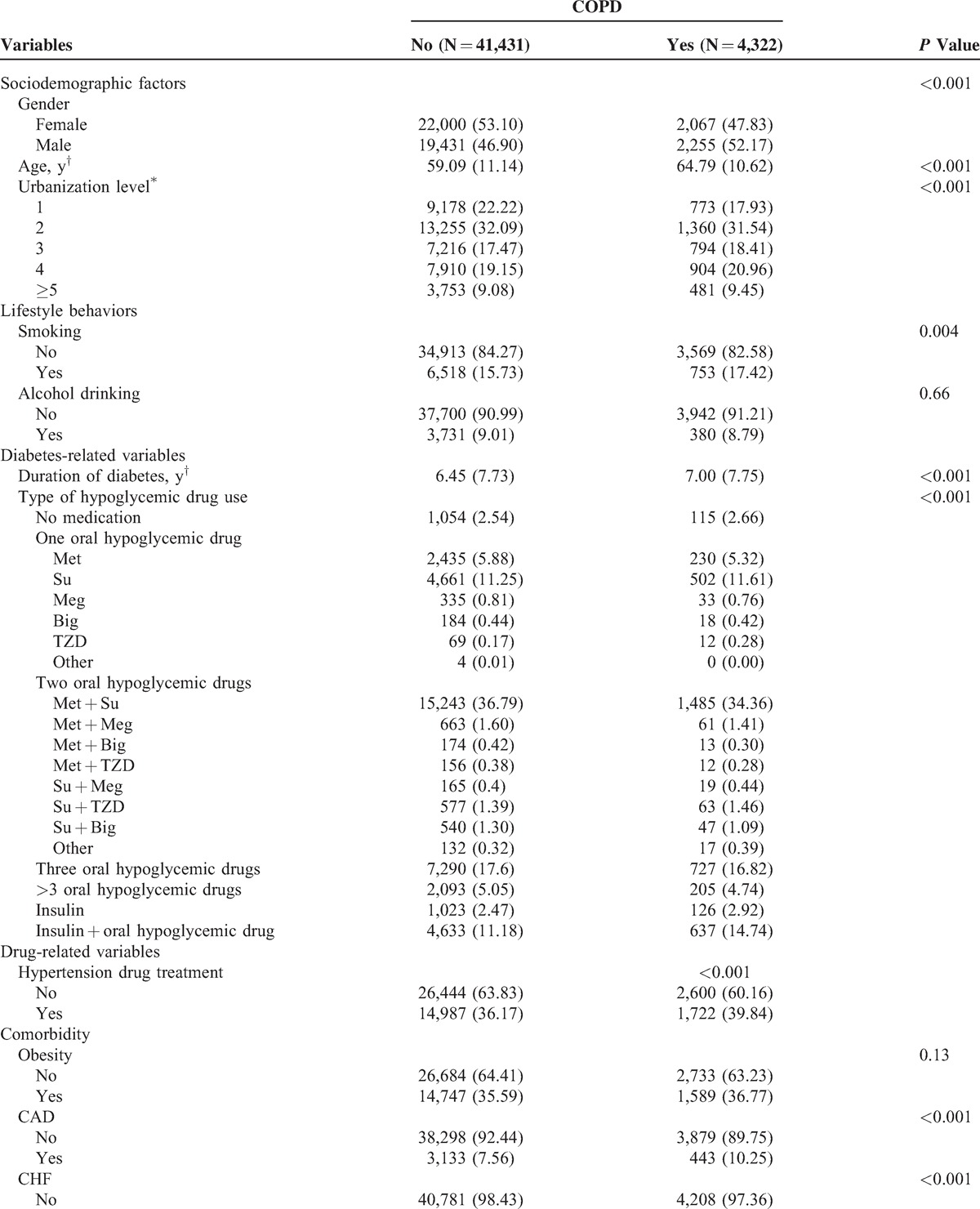
Comparisons of Baseline Sociodemographic Factors, Lifestyle Behaviors, Diabetes-Related Variables, Drug-Related Variables, and Comorbidity According to Chronic Obstructive Pulmonary Disease Incidence in Patients With Type 2 Diabetes Enrolled in the National Diabetes Care Management Program, Taiwan (n = 45,753)

**TABLE 1 (Continued):**
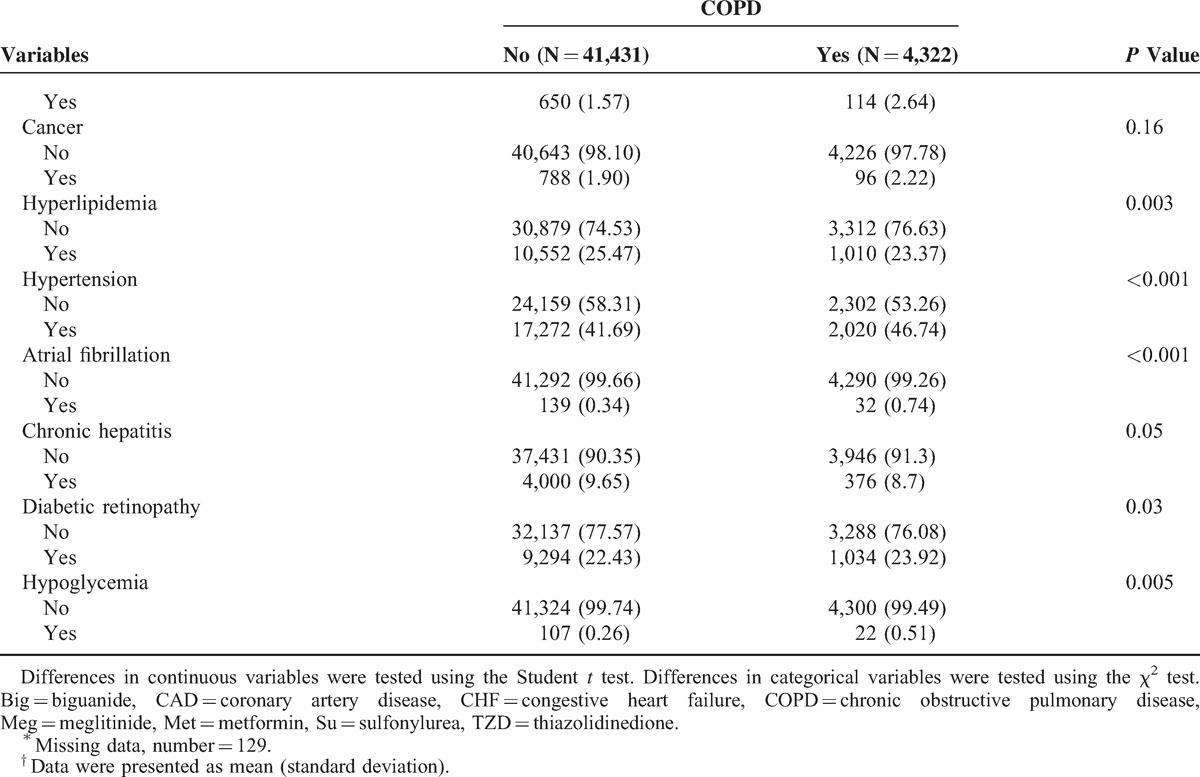
Comparisons of Baseline Sociodemographic Factors, Lifestyle Behaviors, Diabetes-Related Variables, Drug-Related Variables, and Comorbidity According to Chronic Obstructive Pulmonary Disease Incidence in Patients With Type 2 Diabetes Enrolled in the National Diabetes Care Management Program, Taiwan (n = 45,753)

Table 2 shows the HRs of COPD among the patients grouped by HbA1c levels. Compared with patients exhibiting HbA1c levels of 6%–7%, the age- and gender-adjusted HRs of COPD in patients with HbA1c levels of <6%, 8%–9%, 9%–10%, and ≥10% were 1.21 (95% CI 1.07–1.36), 1.18 (1.07–1.30), 1.25 (1.12–1.39), and 1.46 (1.33–1.60), respectively. When considering lifestyle factors, urbanization level, comorbidities, and complications, the effect of HbA1c was slightly attenuated, but remained statistically significant (*P* < 0.05) for HbA1c levels of <6% and ≥10%. Patients with HbA1c levels <6% and ≥10% exhibited a higher risk of COPD (adjusted HR: 1.19 [1.06–1.34] and 1.19 [1.06–1.32], respectively) than did patients with HbA1c levels of 6%–7%. We observed a U-shaped relationship between the HbA1c categories and COPD occurrence.

**TABLE 2:**
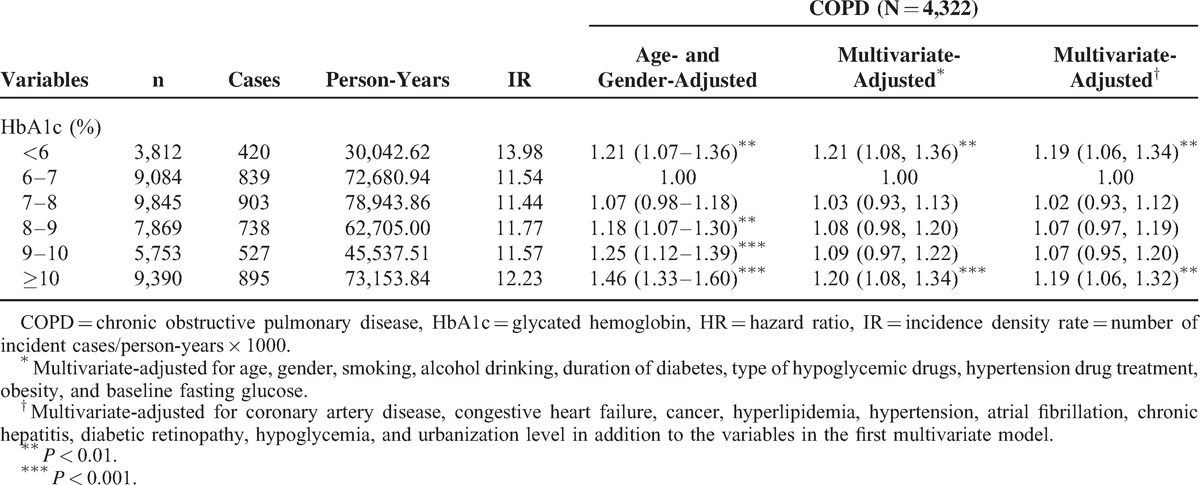
HRs of COPD According to Different HbA1c in Diabetic Patients Enrolled in the National Diabetes Care Management Program, Taiwan (n = 45,753)

**FIGURE 3:**
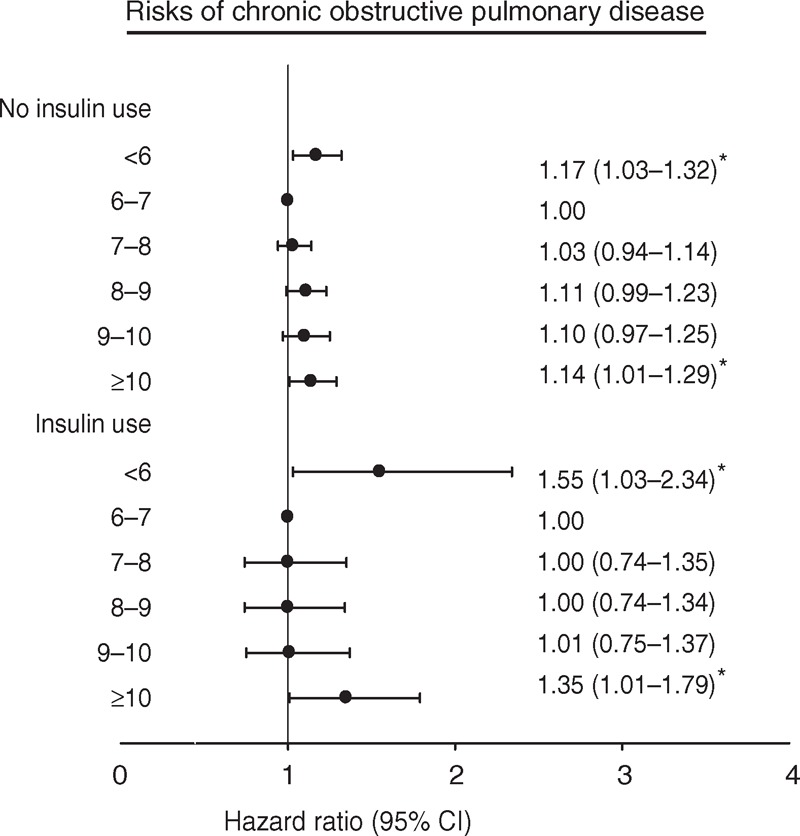
Adjusted HRs of COPD in baseline HbA1c regarding insulin use (no or yes). HRs adjusted for age, gender, smoking, alcohol drinking, duration of diabetes, type of hypoglycemic drugs, hypertension drug treatment, obesity, baseline fasting glucose, coronary artery disease, congestive heart failure, cancer, hyperlipidemia, hypertension, atrial fibrillation, chronic hepatitis, diabetic retinopathy, hypoglycemia, and urbanization level. CI = confidence interval, COPD = chronic obstructive pulmonary disease, HbA1c = glycated hemoglobin, HR = hazard ratio. ^∗^*P* < 0.05.

Sensitivity analyses were conducted to rule out the potential bias resulting from the existence of comorbidities by excluding patients with stroke, cardiovascular disease (CVD), diabetic ketoacidosis, hyperglycemic hyperosmolar nonketotic coma, liver disease, and nephropathy (n = 31,563 for sensitivity analysis). Similar significant (*P* < 0.05) HRs for COPD were found among patients with HbA1c levels <6% and ≥10% (1.16 [1.00–1.34] and 1.25 [1.09–1.43], respectively). The results of further stratification by insulin use (Figure 3) were consistent with those of the primary analysis.

## DISCUSSION

In the present study, we observed a U-shaped association between HbA1c levels and COPD incidence. The risk of COPD was higher among people with HbA1c levels below 6% and above or equal to 10.0% than among those with HbA1c levels of 6%–7%. Excluding people with existing chronic diseases and acute complications, data stratification by insulin use still revealed a similar U-shaped association.

Patients with type 2 diabetes exhibit HbA1c levels above 10% or below 6%, which are associated with a substantially increased risk of COPD. In contrast to data on the incidence of microvascular and macrovascular outcomes,^15–17^ the relationship between glycemic control and risk of COPD in patients with type 2 diabetes has been rarely examined. To the best of our knowledge, the potential harms of an improved glycemic control on the risk of COPD in patients with type 2 diabetes have yet to be determined. Previous studies have revealed that an intensive glycemic control reduces the risk of CVD morbidity and mortality.^17,18^ However, recent trials have suggested that HbA1c target levels below 6.5% result in an increased CVD mortality,^17^ but not in a reduction or increase in CVD events^19–21^ in patients with type 2 diabetes. Our findings regarding an increased risk of COPD in relation to both low and high levels of HbA1c have the same clinical implication as these published results.

COPD primarily affects the lungs, but also produces adverse consequences, including hospitalization or critical care.^22^ A COPD diagnosis is based on a history of persistent progressive symptoms and a confirmatory spirometric test.^23^ An unbalanced glycometabolism might lead to the development of COPD in several ways. Inflammatory markers increase in patients with type 2 diabetes,^24–27^ which may result in endothelial dysfunction that would contribute to airflow obstruction and reduced lung function.^28^ This may explain the chronic state of inflammation observed in patients with COPD, which resembles that by which peripheral airway inflammation leads to airflow obstruction in asthma.^29^ The other possible explanation is that the lung may be a target organ for diabetes. Postmortem histopathological studies have revealed that the lung is indeed a target organ for diabetic microangiopathy, in both type 1 and type 2 diabetes. Additional evidence reveals a peripheral airway dysfunction in type 1 diabetes in the absence of cigarette consumption, allergies, or other common causes of airflow obstruction. Clinical observation indicates that diabetes markedly reduces the respiratory pump capacity.^30^ This finding was supported by an animal study that demonstrated that severe impairment of the diaphragmatic function was induced by diabetes, resulting in a substantially decreased respiratory muscle capacity to manage elevated workloads.^30^ In addition, a human study provided evidence that diabetic polyneuropathy is associated with a substantially impaired respiratory neuromuscular function in patients with type 2 diabetes.^31^

We observed that type 2 diabetes patients with HbA1c levels <6% exhibited a higher risk of developing COPD, which may be due to the higher likelihood of hypoglycemia. In our study, the multivariate-adjusted HRs of COPD in patients with HbA1c levels of <6% was 1.78 (95% CI 1.14–2.81) compared with those in patients exhibiting HbA1c levels of 7%–8%. There are some possible mechanisms by which hypoglycemia could increase risk of subsequent COPD in patients with type 2 diabetes. COPD is a condition that is characterized by irreversible airway obstruction, the result of a chronic inflammatory process in the lungs. The number of apoptotic alveolar epithelial cells in the lung tissue has been associated with chronic airway obstruction.^32,33^ Vascular endothelial growth factor (VEGF) increases survival and inhibits apoptosis in endothelial cells^34^ and is abundantly expressed in rat lung tissue.^35^ Apoptosis through VEGF signaling has been hypothesized to be a mechanism for the development of emphysema and COPD. Low levels of VEGF have been observed in sputum from patients with COPD, and VEGF sputum levels were inversely correlated with airflow limitation assessed as forced expiratory volume in 1 second.^36^ In a recent study, it has been shown that hypoglycemia reduces VEGF-A production, which specifically acts on endothelial cells and has various effects, including endothelial cell growth and inhibiting apoptosis.^37^ The other possible mechanism is that hypoglycemia increases platelet aggregation and fibrinogen formation,^38^ and this may accelerate vascular compromise in the lung. Severe COPD is today recognized as a systemic disease with manifestations from organ systems other than the lungs and airways. A number of studies have shown that higher levels of markers of systemic inflammation including serum levels of fibrinogen^39–42^ are increased in patients with COPD compared with those in healthy control subjects. Furthermore, serum levels of fibrinogen are related to the annual decline in lung function assessed as forced expiratory volume in 1 second (FEV1) in COPD.^43^

Our findings are valuable because computerized reporting of HbA1c is used to assist physicians in identifying type 2 diabetes patients at high risk of adverse outcomes. Current guidelines for glucose control based on HbA1c do not explicitly consider low HbA1c levels. Type 2 diabetes patients treated with HbA1c levels lower than 6% might be harmed because of the excess risk of COPD. The results of the present study suggest that health professionals should also pay attention to type 2 diabetes patients with extremely low or high HbA1c levels.

## LIMITATIONS AND STRENGTHS OF THIS STUDY

This study exhibits several limitations. First, we used only 1 HbA1c measurement. Therefore, we could not evaluate the effect of changes in HbA1c levels over time on the risk of COPD. Second, our study population comprised patients with type 2 diabetes, who were enrolled in the NDCMP. To evaluate the representativeness of this study population, we examined the demographic characteristics of the patients in this study and those enrolled in the NHI program by comparing their age and sex structure, and observed similar distributions. The nondifferential distributions in age and sex indicate that our findings can be generalized to the population with type 2 diabetes in Taiwan. Finally, we adjusted for several lifestyle behaviors and complications, which allowed us to minimize the effect of potential confounders. However, we still could not exclude the possibility of residual confounding.

This study also exhibited several strengths. First, the study sample consisted of numerous diabetic patients. Second, NDCMP used a standardized procedure for collecting data and available information on numerous potential confounding factors. Finally, the study sample was obtained from a national-based population of type 2 diabetes, contributing to the generalizability of the obtained results.

Both extreme HbA1c levels were related to an increased risk of COPD among adult Chinese people with type 2 diabetes in Taiwan. Excluding patients with existing chronic diseases, this U-shaped association remained when we examined the data by insulin use. The consistency of our findings supports the argument that extreme HbA1c levels increase the risk of COPD. Future studies should be conducted to determine how to meet the recommended HbA1c targets that could improve patient outcomes, without posing risks to this population.
